# Neurosteroid Modulation of Synaptic and Extrasynaptic GABA_A_ Receptors of the Mouse Nucleus Accumbens

**DOI:** 10.3390/biom14040460

**Published:** 2024-04-09

**Authors:** Scott J. Mitchell, Grant D. Phillips, Becks Tench, Yunkai Li, Delia Belelli, Stephen J. Martin, Jerome D. Swinny, Louise Kelly, John R. Atack, Michael Paradowski, Jeremy J. Lambert

**Affiliations:** 1 Division of Cellular & Systems Medicine, School of Medicine, Medical Sciences Institute, Dundee University, Dow Street, Dundee DD1 5HL, UK; scott.mitchell@kcl.ac.uk (S.J.M.); gphillips001@dundee.ac.uk (G.D.P.); btench001@dundee.ac.uk (B.T.); yli002@dundee.ac.uk (Y.L.); d.belelli@dundee.ac.uk (D.B.); s.martin@dundee.ac.uk (S.J.M.); 2 School of Pharmacy & Biomedical Sciences, St. Michael’s Building, University of Portsmouth, White Swan Road, Portsmouth PO1 2DT, UK; jerome.swinny@port.ac.uk (J.D.S.); l.kelly@soton.ac.uk (L.K.); 3 Main Building, Medicines Discovery Institute, Park Place, Cardiff University, Cardiff, CF10 3AT, UK; atackj@cardiff.ac.uk (J.R.A.); paradowskim@cardiff.ac.uk (M.P.)

**Keywords:** allopregnanolone, brexanolone, zuranolone GABAA receptor, tonic inhibition, phasic inhibition, nucleus accumbens

## Abstract

The recent approval of formulations of the endogenous neurosteroid allopregnanolone (brexanolone) and the synthetic neuroactive steroid SAGE-217 (zuranolone) to treat postpartum depression (PPD) has encouraged further research to elucidate why these potent enhancers of GABA_A_R function are clinically effective in this condition. Dopaminergic projections from the ventral tegmental area (VTA) to the nucleus accumbens are associated with reward/motivation and brain imaging studies report that individuals with PPD show reduced activity of this pathway in response to reward and infant engagement. However, the influence of neurosteroids on GABA-ergic transmission in the nucleus accumbens has received limited attention. Here, we investigate, in the medium spiny neurons (MSNs) of the mouse nucleus accumbens core, the effect of allopregnanolone, SAGE-217 and other endogenous and synthetic steroids of interest on fast phasic and tonic inhibition mediated by synaptic (α1/2βγ2) and extrasynaptic (α4βδ) GABA_A_Rs, respectively. We present evidence suggesting the resident tonic current results from the spontaneous opening of δ-GABA_A_Rs, where the steroid-enhanced tonic current is GABA-dependent. Furthermore, we demonstrate local neurosteroid synthesis in the accumbal slice preparation and reveal that GABA-ergic neurotransmission of MSNs is influenced by an endogenous neurosteroid tone. Given the dramatic fluctuations in allopregnanolone levels during pregnancy and postpartum, this neurosteroid-mediated local fine-tuning of GABAergic transmission in the MSNs will probably be perturbed.

## 1. Introduction

In the 1980s, neurosteroids, typified by allopregnanolone, were identified as endogenous, potent, positive allosteric modulators (PAMs) of the GABA_A_R [1]. This discovery encouraged exploration of the therapeutic potential of endogenous and synthetic neuroactive steroids including use as anticonvulsants, analgesics, anxiolytics, and antidepressants [2,3,4,5,6,7,8,9,10]. Recently, this potential was realised with the FDA approval of allopregnanolone (brexanolone) and a synthetic derivative SAGE-217 (zuranolone) to treat PPD [2,3,4,5,6,7,8,9,11], reinvigorating interest in targeting GABA_A_Rs in relevant neural pathways for the treatment of depressive disorders [2,3,4,5,6,7,8,9,11].

The mesolimbic dopamine system comprises a vital component of the reward system and is implicated in disrupted mood and anhedonia, integral components of depressive disturbances [12,13,14]. The ventral tegmental area is a key hub sending dopaminergic innervation to the prefrontal cortex, amygdala, and the nucleus accumbens. These dopaminergic projections are implicated in maternal behaviours associated with reward and motivation for both rodents and humans [15]. Furthermore, human brain imaging studies report that those with PPD exhibit reduced activation of these VTA-driven pathways in response to reward and to infant offspring engagement [15,16].

In the forced swim test, the local administration of allopregnanolone into the nucleus accumbal core of rats reduced their immobility and increased climbing, suggesting an antidepressant-like effect of the steroid [17]. In mice, systemic allopregnanolone produced conditioned place preference (CPP), implying that the steroid was perceived as rewarding [18]. By contrast, the intracerebroventricular (ICV) infusion of allopregnanolone in rats produced conditioned place aversion (CPA) [19]. Systemic administration of the neuroactive steroid ganaxolone also produced CPA, an effect dependent on δ-GABA_A_R subunit expression [20]. Neurochemical studies reported low doses of allopregnanolone administered ICV increased dopamine release in the nucleus accumbens [21] an effect potentially resulting from disinhibition of the VTA GABA-ergic interneurons (see below). However, greater doses of the steroid were reported to decrease dopamine release, suggesting a dose-dependent bimodal relationship [22]. Adding complexity, the effect of allopregnanolone on evoked dopamine release appears dependent on the sex and oestrous cycle stage [23].

Investigation of the effect of neurosteroids upon GABA-ergic transmission mediated by GABA_A_R subtypes in the VTA–accumbens pathway is limited. In the mouse VTA, both GABA-ergic and dopaminergic neurons express synaptic GABA_A_Rs that mediate fast phasic inhibitory postsynaptic currents (IPSCs) and extrasynaptic δ-GABA_A_Rs that mediate a tonic current [20]. The acute application of ganaxolone (500 nM) preferentially enhanced the tonic current of the GABA-ergic interneurons vs. that of the dopaminergic neurons with little effect on the synaptic GABA_A_R-mediated response of the GABA-ergic interneurons [20]. Such neuronal selectivity could produce disinhibition of the dopamine neurons, to potentially increase dopamine release from their terminal fields, e.g., the nucleus accumbens. In support of this suggestion, a recent study demonstrated cocaine to induce GABA release from astrocytes in the VTA, thereby increasing the tonic current of the GABA-ergic interneurons, which consequently increased firing of the dopamine projection neurons to the nucleus accumbens [24].

Most neurons (~95%) in the nucleus accumbens are GABA-ergic. We reported that mouse MSNs express synaptic α1- and α2-βγ2 GABA_A_Rs that mediate fast phasic inhibition and extrasynaptic α4βδ GABA_A_Rs that underpin tonic inhibition [25,26,27]. Extrasynaptic δ-GABA_A_Rs are considered an important target in mediating the effects of allopregnanolone in PPD/MDD [2,4,5,6,9] and in influencing behaviours induced by chronic stress [28]. Intriguingly, we found for dopamine 1 receptors (D1Rs) expressing MSNs that their selective agonist activation, or indirect activation by amphetamine, produced a rapid increase in the MSN tonic current mediated by α4βδ GABA_A_Rs [25]. However, adding complexity dopamine 2 receptor (D2R) activation of D2R-expressing MSNs produced a relatively slow decrease in the tonic current [25].

Studies on the rewarding effects of drugs such as ethanol and cocaine suggest an important role for accumbal δ-GABA_A_Rs in the behavioural effects of neurosteroids. In rats, viral siRNA suppression of δ or α4 expression in the nucleus accumbens reduced ethanol intake [29,30]. Complementing these observations, we reported that mice carrying a spontaneous single mutation of the β1 subunit possessed a greatly augmented MSN tonic current and presented with an increased ethanol preference/intake [27]. Furthermore, activation of this tonic current by intra-accumbal administration of the δ-GABA_A_R preferring agonist THIP (gaboxadol) reduced cocaine facilitation of CPP, an effect absent in mice with a selective deletion of the α4 gene in D1R-expressing MSNs [25]. For D2R-expressing MSNs, α4-GABA_A_Rs play a critical role in the expression of the reinforcing properties of discrete reward-conditioned cues [31]. Collectively, these findings are consistent with an important role for α4βδ GABA_A_Rs in the modulation of the reward pathway, but despite its behavioural relevance the effect of neurosteroids upon the accumbal tonic conductance is not known.

Given the efficacy of allopregnanolone (brexanolone) and SAGE-217 (zuranolone) in treating PPD, we investigated the effects of these clinically important steroids on phasic and tonic inhibition of mouse accumbal MSNs in comparison to related endogenous and synthetic steroids of interest. We discovered that the resident constitutive tonic current of MSNs results from the spontaneous opening of GABA_A_Rs, whereas the steroid-enhanced tonic current is GABA-dependent. Intriguingly, given the dynamic changes in allopregnanolone levels during pregnancy and postpartum, we demonstrate that GABA-ergic neurotransmission in accumbal MSNs is under the influence of an endogenous neurosteroid tone and provide evidence that suggests the nucleus accumbens can locally synthesise GABA_A_R-active neurosteroids.

## 2. Materials and Methods

The methods have been previously reported and are described here with minor modifications [25].

### 2.1. Animals

For electrophysiology, C57/BL6J adult male wildtype mice were group-housed at the University of Dundee. The mice had ad libitum food and water. A 12 h light/dark cycle was used with lights on at 7:00 a.m.; the temperature and humidity were controlled at 21 ± 2 °C and 50 ± 5%, respectively. The mice were humanely killed by cervical dislocation in accordance with Schedule 1 of the UK Government Animals (Scientific Procedures) Act of 1986 and subject to ethical review by the “Welfare and Ethical Use of Animals Committee” of the University of Dundee on 13 October 2014 and 23 August 2020. For studies of immunohistochemistry, adult C57BL/6 mice, both male and female, were used. All mice were bred in-house at the University of Portsmouth animal facility and housed under standard conditions, including 25 °C temperature 12 h light/dark cycle (lights turned on at 7:00 a.m.) and with ad libitum access to food and water. All efforts were made to utilise only the minimum number of mice necessary to produce reliable scientific data. All procedures involving animal experiments were approved by the Animal Welfare and Ethical Review Body of the University of Portsmouth, October 2019, and were performed by a personal license holder, under a Home Office-issued project license number PP4887767, issued on 10 January 2020, in accordance with the UK Government Animals (Scientific Procedures) Act, of 1986.

### 2.2. Electrophysiology Brain Slice Preparation

Following cervical dislocation, coronal brain slices (300 µm) containing the nucleus accumbens were prepared using a Leica VT 1200S vibratome, fitted with a ceramic blade (EF-INZ10, Cadence Blades). Slicing was conducted in an oxygenated ice-cold solution containing (mM): 140 potassium gluconate, 10 HEPES (N-2-hydroxyethylpiperazine-N-2-ethane sulfonic acid), 15 sodium gluconate, 0.2 EGTA (ethylene glycol-*bis* (β-aminoethyl)-N,N,N′,N′-tetra acetic acid) and 4 NaCl, pH 7.2 with KOH, 300–310 mOsm. Slices were allowed to rest for a minimum of 1 h in an oxygenated extracellular solution (ECS) at room temperature containing (mM) the following: 126 NaCl, 26 NaHCO_3_, 2.95 KCl, 1.25 NaH_2_PO_4_, 2 MgCl_2_, 2 CaCl_2_, 10 glucose, 308–312 mOsm. For experiments using a drug/steroid incubation protocol, the slices were maintained with the compound in oxygenated ECS at room temperature for 2 h.

### 2.3. Electrophysiology Recording

MSNs in the core region of the nucleus accumbens were visually identified using an Olympus BX51WI microscope fitted with DIC/infrared optics. Whole-cell voltage-clamp recordings were carried out at a holding potential Vh of −60 mV in ECS containing strychnine (1 µM), tetrodotoxin—TTX (1 µM) and kynurenic acid (2 mM) and maintained at 34–35 °C. For experiments with compound preincubation, the drug at the same concentration was also included in the circulating ECS. Patch electrodes (tip resistance 3–5 MΩ) contained an intracellular solution (mM) including the following: 135 CsCl, 10 HEPES, 10 EGTA, 1 CaCl_2_, 1 MgCl_2_, 2 MgATP and 5 QX-314, pH 7.2–7.3 with CsOH, 300–308 mOsm. Data were acquired using Axopatch 200B amplifier (Axon Instruments, Burlingame, CA, USA) and digitised using a NI USB-6221 A/D converter. Data were low-pass filtered at 2 kHz and sampled at 10 kHz. WinEDR and WinWCP programs (J. Dempster, University of Strathclyde) were used for acquisition and analysis. Recordings showing a >20% change in series resistance during the experiment were discarded.

### 2.4. Drugs

Kynurenic acid (Sigma-Aldrich, Dorset, UK) was directly dissolved in ECS on the day of use. The γ-cyclodextrin (Sigma-Aldrich) was directly dissolved in the intracellular solution. Stock solutions of strychnine hydrochloride (Sigma), TTX, bicuculline methiodide and gabazine hydrobromide (all HelloBio, Bristol, UK), were made in distilled H_2_O. 5α-pregnane-3α,20α-diol [5α-pregnanediol] (SantaCruz Bio PubChem CID 164,674 (566-58-5), ganaxolone (Bio-Techne Tocris, Oxford, UK), 5α-pregnane,3,20-dione [5α-dihydroprogesterone] (Sigma), SAGE-217 (MedChem Express, Monmouth Junction, NJ, USA), Co2-1970 (synthesised at MDI, Cardiff University), 5α-pregnane-3α-ol-20-one [allopregnanolone] (Tocris), DS2 (Tocris) and picrotoxin (Sigma-Aldrich) stocks were made in DMSO (100%). The maximum final concentration of DMSO in ECS was 0.3%.

### 2.5. Electrophysiology Data Analysis

The change in holding current was determined by calculating the mean current over 102.4 ms epochs for 30 s. Epochs were visually inspected and removed if unstable or containing miniature inhibitory postsynaptic currents (mIPSCs). Two control 30 s sections of holding current were obtained, at least 5 min apart, and averaged. A drug section was defined once stability of the holding current in the presence of the drug had been achieved and was determined as before for a single 30 s portion of the control recording. The change in holding current was taken as the difference between the control and drug period. The mIPSCs were initially detected using a rate of rise of 50–70 pA ms^−1^ which aided their identification particularly given the increased membrane noise associated with drug-enhanced tonic currents. All mIPSCs were then visually inspected. A minimum of 30 mIPSCs per neuron were used for analysis of peak amplitude, rise time (10–90%, <1 ms), and decay time. A biexponential function (y(t) = A_1_e^(−t/τ1)^ + A_2_e^(−t/τ2)^) was fitted (98-5% of peak amplitude) to the decay phase of averaged mIPSCs. In this function, t is time, A is amplitude, and τ is the decay time constant. A weighted decay constant τ_w_ was calculated, accounting for the contributions of the fast and slow components of the decay. This is shown as τ_w_ = τ_1_P_1_ + τ_2_P_2_, where τ represents the decay time constant, and P represents the proportion of the decay each component contributes.

### 2.6. Electrophysiology Statistical Analysis

Data are reported as mean ± standard error of the mean (SEM). Data were assessed for a normal distribution assumption using a one-sample Kolmogorov–Smirnov test. Statistical significance was determined by Student’s *t*-tests (independent and paired samples). Nonparametric tests included a Mann–Whitney U test and a Wilcoxon signed-rank test (independent and paired samples, respectively). All statistical analysis was conducted in IBM SPSS, Version 28.0. Histograms and representative traces were produced in Origin 2021b and Inkscape 1.3.

### 2.7. Immunohistochemistry Studies

Anaesthesia was induced with isoflurane and maintained with pentobarbitone (Pentoject^®^, Product Code: XVD133, Animalcare, York, UK) at a dose of 1.25 mg/kg I.P. of bodyweight. Mice were transcardially perfused, first with 0.9% saline for 1 min, followed by a fixative solution containing 1% formaldehyde, in 0.1 M phosphate buffer (PB), pH 7.4, for 10 min. Brains were then dissected and post-fixed in a formaldehyde solution overnight at room temperature, then washed with 0.1 M PB solution before sectioning with a Vibratome (Leica Microsystems, Wetzlar, Germany). Tissue sections (60 µm) were stored in 0.1 M PB containing 0.05% sodium azide. For free-floating immunohistochemical experiments, tissue sections were first subjected to proteolytic antigen unmasking using a method previously described [32]. Briefly, the tissue sections were incubated at 37 °C for 10 min in 0.1 M PB in a shaking incubator. This treatment was followed by 10 min at 37 °C in 0.2 M HCl containing 1 mg/mL pepsin (catalogue number P6887-250MG, Sigma Aldrich, UK), after which they were washed for 3 × 10 min in Tris-buffered saline containing 0.3% triton (TBS-Tx). To minimise non-specific antibody binding, tissue sections were incubated with 20% normal horse serum (catalogue number S-2000-20, Vector Laboratories, Burlingame, CA, USA), diluted with TBS-Tx, for 2 h at room temperature. Tissue sections were then incubated overnight at 4 °C in a cocktail of primary antibodies consisting of goat anti DARPP-32 (Santa Cruz, catalogue number 8483); mouse anti neuroligin 2 (Synaptic Systems, catalogue number 129 511), and either rabbit anti GABA_A_R β1 (Synaptic Systems, catalogue number 224 703), rabbit anti GABA_A_R β2 (Synaptic Systems, catalogue number 224 803) or rabbit anti GABA_A_R β3 (Synaptic Systems, catalogue number 224 403). Following washing the next day in TBS-Tx, the sections were then incubated with secondary antibodies for 2 h at room temperature. The following secondary antibodies (and their concentrations) were used; Alexa Fluor^®^ 488 AffiniPure™ Donkey Anti-Rabbit IgG, 1:1000 (Jackson ImmunoResearch, catalogue number 711-545-152), Cy™3 AffiniPure™ Donkey Anti-Mouse IgG, 1:1000 (Jackson ImmunoResearch, catalogue number 715-165-150). Alexa Fluor^®^ 647 AffiniPure™ Donkey Anti-Goat IgG, 1:1000 (Jackson ImmunoResearch, catalogue number 705-605-003). Sections were then washed for 3 × 10 min. in TBS-Tx and mounted onto glass slides in Vectashield Antifade Mounting Medium (catalogue number H-1000-10, Vector Laboratories, Burlingame, CA, USA).

### 2.8. Immunohistochemistry Image Acquisition

The tissue sections were examined with a LSM710 microscope (Zeiss, Oberkochen, Germany) using a Plan Apochromatic 63 x DIC oil objective (NA 1.4, pixel size 0.13 μm) and the following lasers and setting. argon, 488 nm, 2% power; HeNe1 (543 nm), 2% power and HeNe2 (633 nm). Images were acquired using sequential acquisition of the individual channels to avoid crosstalk between fluorophores, with the pinholes adjusted to one Airy unit for all channels. Z-stacks were used for routine evaluation of the labelling. All images presented represent a single optical section. Only brightness and contrast were adjusted for the whole frame, and no part of a frame was enhanced or modified in any way. They were then exported as TIFF files and figures composed using Adobe Photoshop software Version 25.6 (Adobe Inc., San Jose, CA, USA).

## 3. Results

### 3.1. Phasic and Tonic Inhibition of Medium Spiny Neurons of the Nucleus Accumbens

**Figure 1:**
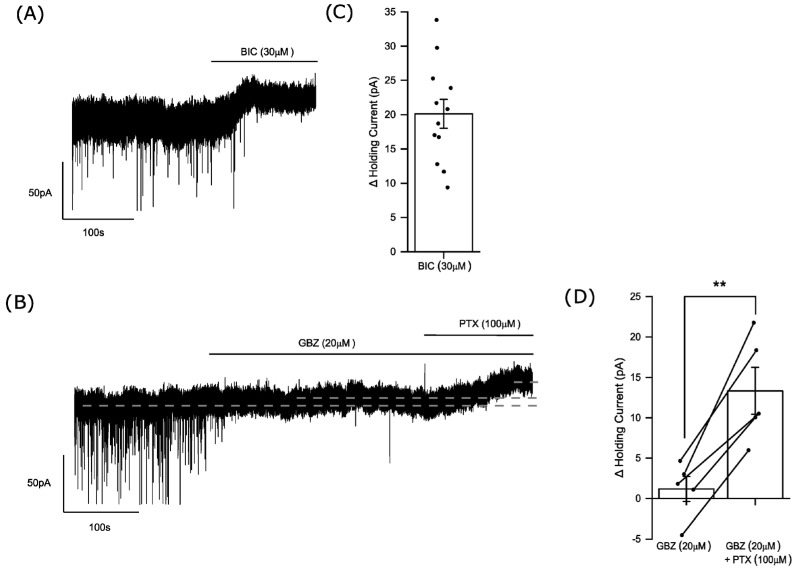
Is the tonic current of accumbal medium spiny neurons mediated by spontaneously open extrasynaptic GABA_A_ receptors? (**A**,**B**) Whole-cell voltage clamp recordings (Vh = −60 mV) of representative current traces obtained from MSNs under control conditions and following application of (**A**) bicuculline (30 µM) and (**B**) gabazine (20 µM), followed by the co-application of gabazine (20 µM) plus picrotoxin (100 µM). The fast downward deflections evident from these recordings result from mIPSCs mediated by synaptic GABA_A_Rs. (**A**) Bicuculline caused a complete inhibition of the mIPSCs and produced a clear outward current. (**B**) Similarly, gabazine completely blocked the mIPSCs but in contrast to bicuculline had no effect on the holding current, although the subsequent co-application of gabazine (20 µM) with picrotoxin (100 µM) produced an outward current. The dotted lines in (**B**) indicate the holding current in control, in the presence of gabazine and in the presence of gabazine plus picrotoxin. (**C**,**D**) Histograms quantifying the effects of (**C**) bicuculline (30 µM) and (**D**) gabazine (20 µM) followed by co-administered picrotoxin (100 µM) on the holding current. (**C**) Bicuculline produced a clear outward current (20 ± 2.1 pA; n = 12 neurons), whereas (**D**) gabazine had no effect on the holding current (1.2 ± 1.6 pA; n = 5 neurons) but the subsequent co-application of picrotoxin with gabazine produced a clear outward current (12 ± 1.9 pA; n = 5 neurons). (**C**,**D**) The columns represent the mean ± SEM with the effect upon individual neurons denoted by the closed black circle symbol. (**D**) the effect on the holding current of gabazine followed by gabazine plus picrotoxin for control MSNs is shown for individual neurons by the line connecting the paired closed symbols of the two columns. (** = *p* < 0.01; paired samples *t*-test). BIC = bicuculline; GBZ = gabazine; PTZ = picrotoxin.

We have reported fast phasic inhibition of adult mouse accumbal medium spiny neurons (MSNs) to be mediated by synaptic α1βγ2 and α2βγ2 GABA_A_Rs and a tonic current mediated by extrasynaptic α4βδ GABA_A_Rs [25,26]. Using the whole-cell voltage-clamp technique (Vh = −60 mV), control mIPSCs exhibited a mean peak amplitude of −77 ± 5 pA, a rise time of 0.50 ± 0.01 ms, and a decay time (τ_w_) of 8.1 ± 0.3 ms (n = 29 neurons). The application of the GABA_A_R antagonist bicuculline completely blocked the mIPSCs and additionally revealed a resident tonic current (20 ± 2.1 pA; n = 12 neurons)—Figure 1A,C. These properties of phasic and tonic inhibition were consistent with our previous reports [25,26].

The GABA_A_R antagonist gabazine (20 µM), in common with bicuculline, completely blocked the mIPSCs. However, in contrast to bicuculline, gabazine had no effect on the tonic current 1.2 ± 1.6 pA (n = 5 neurons)—Figure 1B,D. Recombinant GABA_A_Rs (α1β2γ2) incorporating a mutant β2 subunit that impairs GABA binding, also reduced the apparent affinity for bicuculline and gabazine, suggesting they all act via a common binding site [33]. However, the effect of these antagonists upon activation of GABA_A_Rs by pentobarbital and by alphaxalone suggests that in certain scenarios they do not act as competitive inhibitors but as allosteric modulators, producing conformational changes in the receptor. In neurons exhibiting prominent spontaneous openings of GABA_A_R channels, bicuculline acts as a negative allosteric modulator to close the channel, whereas gabazine is inert in this respect [34,35,36,37]. Therefore, the differential effect of these two orthosteric GABA_A_R antagonists suggests the resident tonic current of accumbal MSNs results from the spontaneous opening of extrasynaptic GABA_A_Rs [34,35,36,37]. Following gabazine perfusion, the subsequent application of the non-competitive GABA_A_R anion channel blocker picrotoxin (100 µM) produced a clear outward current (12.1 ± 1.9 pA; n = 5 neurons)—Figure 1B,D. Note that in the MSNs we did not observe any effect of gabazine to increase the tonic current as reported for dentate gyrus granule neurons [36,37].

**Figure 2:**
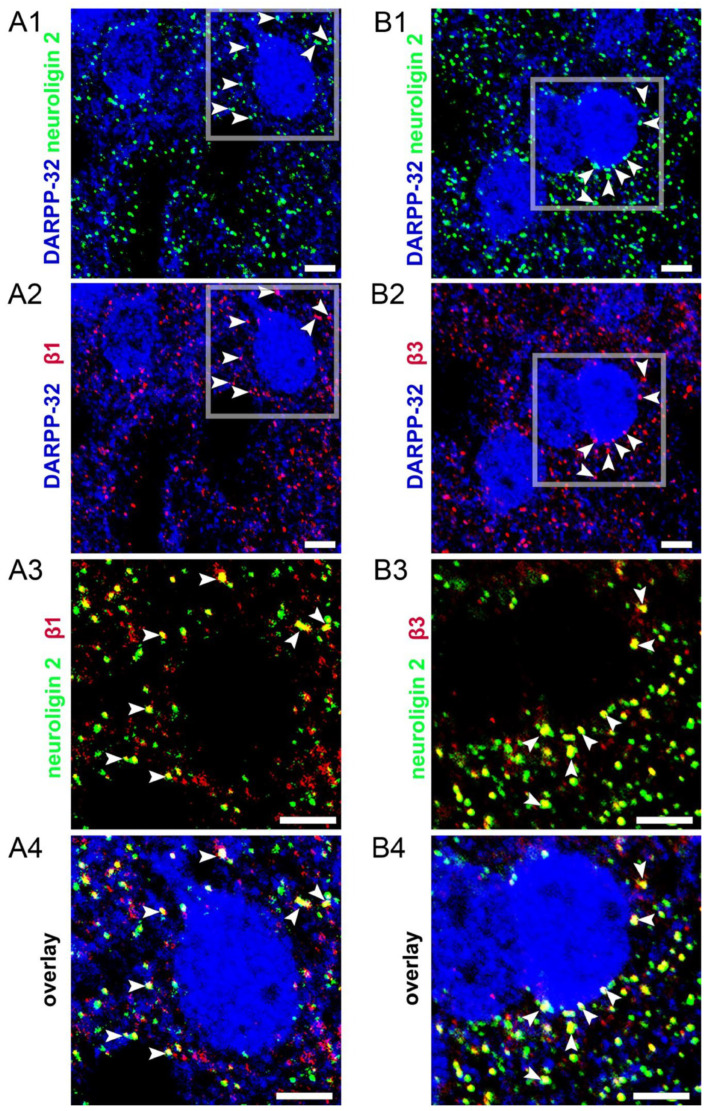
Immunolocalisation of the GABA_A_R β1 and β3 subunits in the medium spiny neurons (MSNs) of the nucleus accumbens. (**A1**) shows the somata and dendritic profiles of MSNs, identified by immunoreactivity for dopamine- and cAMP-regulated phosphoprotein, 32 (DARPP-32) (blue), alongside clusters immunoreactive for the inhibitory synaptic marker protein, neuroligin 2 (green). (**A2**), shows, in the same field of view (FoV), immunoreactivity for the β1, subunit presenting as individual clusters targeted to DARPP-32 immunopositive somatic and dendritic surfaces. (**A3**) is a magnified view of the boxed area in (**A1**,**A2**) showing the strong overlap of clusters immunopositive for the β1 subunit and neuroligin 2, highlighted on a few occasions with arrowheads. (**A4**) is a magnified view of the boxed area in (**A1**,**A2**) showing an overlay of all signals. (**B1**) shows somata and dendritic profiles of MSNs, identified by immunoreactivity for DARPP-32 (blue), alongside clusters immunoreactive for neuroligin 2 (green). (**B2**), shows, in the same FoV, immunoreactivity for the β3 subunit, presenting as individual clusters targeted to DARPP-32 immunopositive somatic and dendritic surfaces. (**B3**) is a magnified view of the boxed area in (**B1**,**B2**) showing the strong overlap of clusters immunopositive for β3 and neuroligin 2, highlighted on a few occasions with arrowheads. (**B4**) is a magnified view of the boxed area in (**B1**,**B2**) showing an overlay of all signals. Scale bars are 5 μm.

Recombinant GABA_A_Rs incorporating the β3 subunit (α4β3δ) exhibit spontaneous gating, whereas equivalent receptors incorporating the β1 or the β2 subunit do not [35]. We previously reported the immunolocalisation of the α1, α2, and α4 GABA_A_R subunits in the mouse accumbal MSNs [25,26]. Here, we built on these expression data to determine which β subunits are expressed by accumbal MSNs of the adult mouse. Immunoreactivity for the β1 subunit presented as individual clusters contacting somatic and dendritic domains of the MSNs, identified by immunoreactivity for the dopamine-and cAMP-regulated phosphoprotein-32 (DARPP-32). Furthermore, numerous β1-immunoreactive clusters colocalised with signals immunopositive for neuroligin 2 (NL 2), a cell adhesion protein that is located exclusively in inhibitory synapses [38] (Figure 2(A1–A4)). In a similar manner, immunoreactivity for the β3 subunit also decorated MSN profiles and overlapped with the majority of NL 2 signals (Figure 2(B1–B4)). Despite assessing a variety of antibodies against the β2 subunit and a variety of reaction conditions, we were unable to detect specific labelling patterns with signals distributed randomly across cytoplasmic, nuclear, and extracellular regions. This suggests that β1 and β3 are the predominant GABA_A_R β subunits expressed in these MSNs. Furthermore, the association of a proportion of their immunoreactive clusters with NL2, and thus putative synaptic domains, suggests they contribute to both synaptic and extrasynaptic GABA_A_R-mediated currents.

### 3.2. The Influence of Allopregnanolone on Phasic and Tonic Inhibition

**Figure 3:**
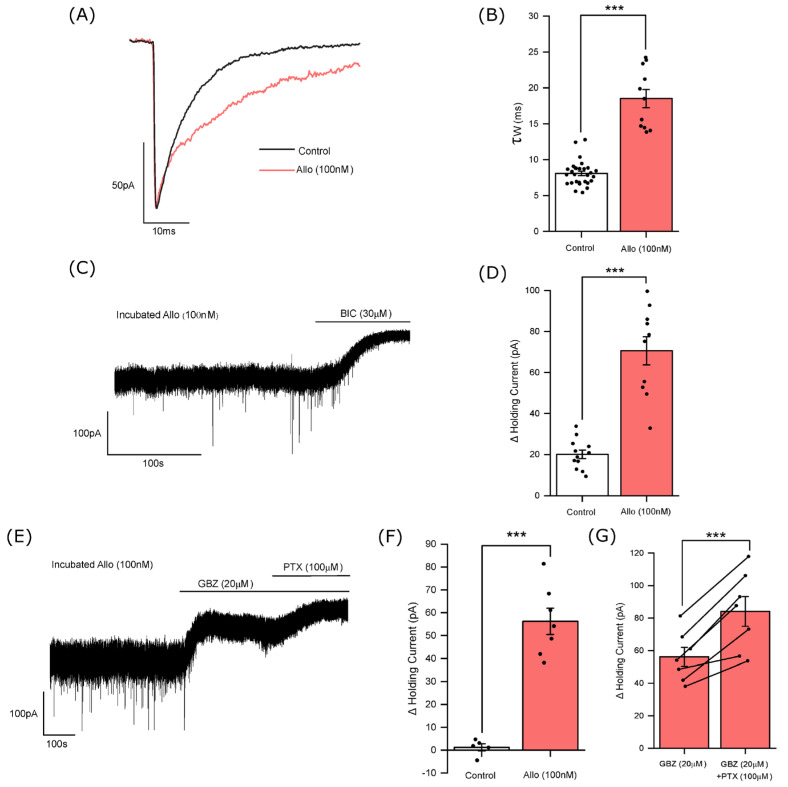
Allopregnanolone (100 nM) greatly enhanced both phasic and tonic inhibition of mouse accumbal medium spiny neurons. (**A**) Illustrated is an averaged mIPSC recorded from an exemplar MSN under control conditions (black trace) and a superimposed averaged mIPSC following preincubation for a minimum of 2 h followed by the continued bath perfusion of 100 nM allopregnanolone (red trace). Note the mIPSC amplitude in the presence of the steroid is normalised to the control mIPSC amplitude to illustrate the effect of the steroid on the mIPSC decay time. (**B**) A histogram illustrating the effect of allopregnanolone (100 nM) upon the mIPSC decay time (τ_w_ ms). Each column represents the mean ± SEM of the mIPSC decay time, with the value for individual neurons denoted by the closed black circle symbol. Treatment with allopregnanolone significantly prolonged the mIPSC decay time constant (control τ_W_ = 8.1 ± 0.3 ms; n = 29 neurons; allopregnanolone τ_W_ = 19 ± 1.3 ms; n = 11 neurons (*** *p* < 0.001, independent samples *t*-test). (**C**,**D**) The effect of allopregnanolone on the tonic current. (**C**) An exemplar trace illustrating the large outward current produced by the application of bicuculline (30 µM) to an MSN preincubated and then subsequently continuously perfused with allopregnanolone (100 nM). (**D**) A histogram illustrating the magnitude of the outward current produced by bicuculline for control MSNs and for neurons treated with allopregnanolone (100 nM). The neurosteroid significantly increased the bicuculline-induced outward current (control = 20 ± 2.1 pA; n = 12 neurons; allopregnanolone = 71 ± 6.9 pA, n = 10 neurons, *** *p* < 0.001, independent samples *t*-test). (**E**) An exemplar trace illustrating the holding current of an MSN treated with allopregnanolone (100 nM). In comparison to the control condition, inspection of the trace revealed gabazine (20 µM) to now produce a substantial outward current. The subsequent co-application of picrotoxin (100 µM) with gabazine (20 µM) produced a further outward current. (**F**) A histogram comparing the magnitude of the outward current produced by the application of gabazine (20 µM) to control neurons, with those treated with allopregnanolone (100 nM). This neurosteroid significantly increased the gabazine-induced outward current (control = 1.2 ± 1.6 pA; n = 5 neurons; allopregnanolone = 56 ± 5.8 pA; n = 7 neurons, *** *p* < 0.001, independent samples *t*-test). (**G**) A histogram comparing the magnitude of the outward current in the presence of allopregnanolone (100 nM) produced by gabazine, with that produced by the subsequent application of picrotoxin in the continued presence of gabazine. The co-application of picrotoxin (100 µM) with gabazine (20 µM) produced a significant additional outward current (gabazine = 56 ± 5.8 pA; n = 7 neurons; gabazine + picrotoxin = 84 ± 9.2 pA, n = 7 neurons *** *p* < 0.001; paired samples *t*-test). For the histograms (**D**,**F**,**G**) each column represents the mean ± SEM of the outward current produced by the antagonists, with the effect upon individual neurons denoted by the closed black circle symbol. In (**G**) for allopregnanolone treated neurons the effect on the holding current of gabazine followed by gabazine plus picrotoxin is shown for individual neurons by the line connecting the paired closed symbols of the two columns. BIC = bicuculline; GBZ = gabazine; PTX = picrotoxin; Allo = allopregnanolone.

The acute application of allopregnanolone (100 nM) produced only a small inward current (−11.9 ± 4.1 pA; n = 6 neurons) and with no significant effect on the mIPSC peak amplitude (control = −76 ± 17 pA; allopregnanolone = −73 ± 10 pA), rise time (control = 0.6 ± 0.03 ms; allopregnanolone = 0.6 ± 0.03 ms), or decay time (control τ_w_ = 9.4 ± 1.1 ms; allopregnanolone τ_w_ = 11.8 ± 2.2 ms)—not shown (*p* > 0.05, n = 5 neurons, paired samples *t*-test for all mIPSC parameters). Previous studies reported that the GABA_A_R PAMs etomidate and propofol require several hours to fully equilibrate with rodent brain slice preparations [39,40]. Given the minimal action of acute allopregnanolone (100 nM) on tonic and phasic inhibition, accumbal slices were now incubated for a minimum of 2 h in ACSF containing allopregnanolone (100 nM). Following incubation and continuous perfusion of the slice with allopregnanolone (100 nM), the steroid now greatly prolonged the decay time (τ_w_) of the mIPSCs (control = 8.1 ± 0.3 ms; n = 29 neurons, vs. allopregnanolone = 18.5 ± 1.3 ms (*p* < 0.001, independent samples *t*-test; n = 11 neurons)—(Figure 3A,B) with no significant effect on the mIPSC peak amplitude or rise time (not shown). The incubation protocol additionally significantly increased the tonic current, revealed by bicuculline, *c.f.* control (allopregnanolone = 71 ± 6.9 pA, n = 10 neurons, *p* < 0.001, independent samples *t*-test)—Figure 3C,D.

### 3.3. Allopregnanolone Increases the Tonic Current in a GABA-Dependent Manner

As described above, the control tonic current was gabazine-insensitive (Figure 1B,D). However, following allopregnanolone (100 nM) incubation, gabazine (20 µM) now produced a large outward current (56 ± 5.8 pA; n = 7 neurons *p* < 0.001, independent samples *t*-test)—Figure 3E,F. Additionally, the subsequent co-application of picrotoxin (100 µM) with gabazine produced a significant further outward current (28 ± 4.3 pA; n = 7 neurons; *p* < 0.001, paired samples *t*-test) Figure 3E,G. In the presence of allopregnanolone, the effect of gabazine on the tonic current suggests that the steroid enhanced the effect of ambient GABA levels sufficiently to now gate the extrasynaptic GABA_A_Rs. The additional effect of picrotoxin implies that in the presence of allopregnanolone a component of the tonic current still results from the spontaneous opening of GABA_A_Rs.

### 3.4. DS2 Selectively Enhances the Tonic Current

We had previously shown for accumbal core MSNs obtained from wild type mice that DS2, a δ-GABA_A_R selective PAM [41] increased the tonic current, but in contrast to the allopregnanolone had no effect on the mIPSCs [25]. Furthermore, DS2 had no effect on the magnitude of tonic current of MSNs obtained from δ^−/−^ mice [25]. Given that allopregnanolone is not selective for δ-GABA_A_Rs [42] we now investigated whether in common with allopregnanolone the DS2-enhanced tonic current was gabazine-sensitive.

**Figure 4:**
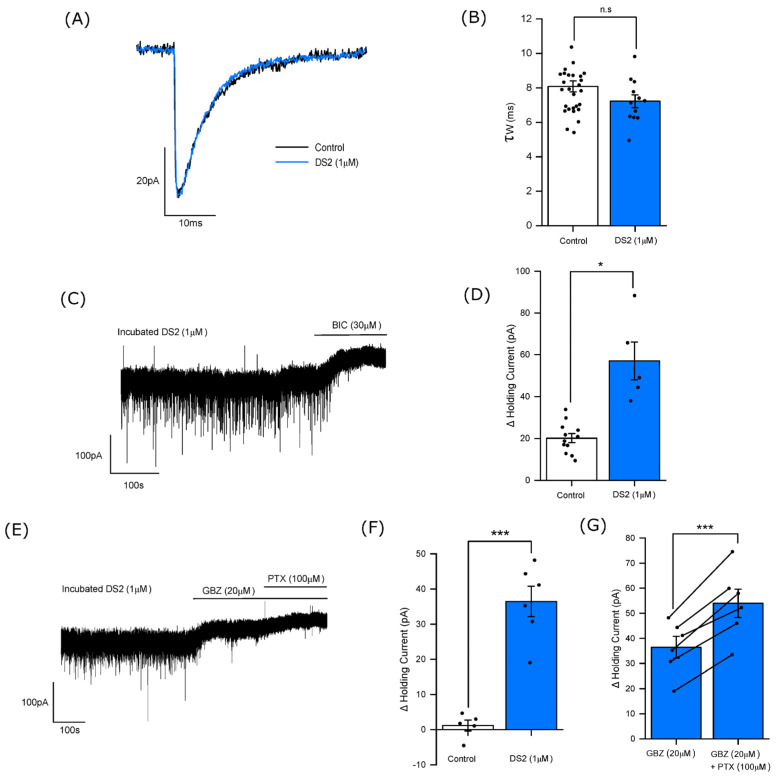
DS2 (1 µM) selectively enhances the tonic current of accumbal medium spiny neurons (MSNs). (**A**) Illustrated is an averaged mIPSC recorded from an exemplar MSN under control conditions (black trace) and a superimposed averaged mIPSC (blue trace) following preincubation and the continued bath perfusion of DS2 (1 µM). Note the mIPSC amplitude in the presence of the steroid is normalised to the control mIPSC amplitude to illustrate the lack of an effect of DS2 on the mIPSC decay time. (**B**) A histogram illustrating that DS2 had no significant (n.s) effect on the mIPSC decay time course (control τ_W_ = 8.1 ± 0.3 ms, n = 29 neurons, DS2 τ_W_ = 7.2 ± 0.4 ms, n = 12 neurons, independent samples *t*-test). (**C**,**D**) The effect of DS2 on the tonic current. (**C**) An exemplar trace illustrating the outward current produced by the application of bicuculline (30 µM) to an MSN preincubated and continuously perfused with DS2 (1 µM). (**D**) A histogram illustrating the magnitude of the outward current produced by the application of bicuculline (30 µM) to control MSNs and to neurons treated with DS2 (1 µM). DS2 significantly increased the bicuculline-induced outward current (control = 20 ± 2.1 pA; n = 12 neurons; DS2 = 57 ± 9.1 pA; n = 5 neurons, * *p* < 0.05, independent samples *t*-test. (**E**) An exemplar trace illustrating the holding current of a MSN treated with DS2 (1 µM). In comparison to the control condition, inspection of the trace revealed gabazine (20 µM) to now produce a substantial outward current. The subsequent co-application of picrotoxin with gabazine produced a further outward current. (**F**) A histogram comparing the magnitude of the outward current produced by the application of gabazine (20 µM) to control neurons, with those treated with DS2 (1µM). DS2 significantly increased the gabazine-induced outward current (control = 1.2 ± 1.6 pA, n = 5 neurons; DS2 = 36 ± 4.3 pA; n = 6 neurons *** *p* < 0.001, independent samples *t*-test). (**G**) A histogram comparing the magnitude of the outward current in the presence of DS2 (1µM) produced by gabazine with that produced by the subsequent application of picrotoxin in the continued presence of gabazine. The co-application of picrotoxin (100 µM) with gabazine (20 µM) produced a significant additional outward current (gabazine = 36 ± 4.3 pA; neurons; gabazine + picrotoxin = 54 ± 5.7 pA, n = 6 neurons *** *p* < 0.001; paired samples *t*-test). For histograms (**D**,**F**,**G**) each column represents the mean ± SEM of the outward current produced by the antagonists, with the effect upon individual neurons denoted by the closed black circle symbol. In (**G**) for DS2 treated neurons the effect on the holding current of gabazine, followed by gabazine plus picrotoxin is shown for individual neurons by the line connecting the paired closed symbols of the two columns. BIC = bicuculline; GBZ = gabazine; PTX = picrotoxin.

In agreement with our previous report [25], the acute application of DS2 (10 µM) produced an inward current (−35 ± 8.2 pA; n = 6 neurons). To provide an appropriate comparison with allopregnanolone we investigated the effect of DS2 preincubation on the tonic current. Preliminary experiments with preincubated DS2 (10 µM for 2 h) produced a greatly increased tonic current (>150 pA) revealed by the application of bicuculline (30 µM). Consequently, subsequent experiments used a tenfold lower concentration of preincubated DS2 (1 µM). Under these conditions, DS2 (1 µM) had no effect on the mIPSC decay time (control τ_w_ = 8.1 ± 0.3 ms; n = 29 neurons; DS2 τ_w_ = 7.2 ± 0.4 ms; n = 12 neurons; *p* > 0.05, independent samples *t*-test)—Figure 4A,B. However, the application of bicuculline (30 µM) revealed a large tonic current (control = 20 ± 2.1 pA; n = 12 neurons; DS2 = 57 ± 9.1 pA; n = 5 neurons *p* < 0.05, independent samples *t*-test)—Figure 4C,D. In common with allopregnanolone, a component of the tonic current in the presence of DS2 (1 µM) was now inhibited by the application of (20 µM) gabazine (control = 1.2 ± 1.6 pA; n = 5 neurons; DS2 = 36 ± 4.3 pA; n = 6 neurons)—Figure 4E,F. Additionally, the subsequent application of picrotoxin (100 µM) produced a further outward current (18 ± 2.3 pA, n = 6 neurons, *p* < 0.001; paired samples *t*-test)—Figure 4E,F. Collectively, the differential effect of the GABA_A_R antagonists in the absence and presence of DS2, implies that this selective δ-GABA_A_R PAM enhanced the tonic current by facilitating the interaction of ambient GABA with δ-GABA_A_Rs. Furthermore, in common with allopregnanolone, following the application of gabazine the additional effect of picrotoxin suggests a component of the total tonic current remains mediated by the spontaneous opening of extrasynaptic GABA_A_Rs.

### 3.5. Endogenous Neurosteroids and Their Local Synthesis Influence Synaptic GABA-Ergic Neurotransmission of Accumbal Medium Spiny Neurons

**Figure 5:**
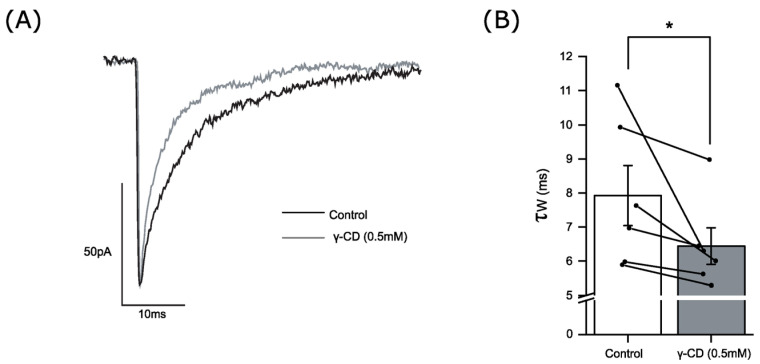
The steroid “scavenger” γ-cyclodextrin decreased the mIPSC decay time of accumbal medium spiny neurons. (**A**) Superimposed averaged mIPSCs from an exemplar MSN recorded briefly after achieving the whole cell recording configuration (0–120 s; black trace) and from the same neuron later in the recording (180–600 s; grey trace). Note the intracellular solution contained γ-cyclodextrin (0.5 mM). The time-dependent decrease in the mIPSC decay time may be caused by the period required for dialysis of the intracellular compartment with γ-cyclodextrin, which then sequesters the endogenous neurosteroid. (**B**) A histogram showing the average mIPSC decay time (τ_w_) in ms, with time from commencing recording (0–120 s) to (180–600 s). The presence of intracellular γ-cyclodextrin (post 180 s) significantly reduced the mIPSC decay time (pre 120 s τ_w_ = 7.9 ± 0.9 ms; post 180 s τ_w_ = 6.4 ± 0.5 ms, n = 6 neurons; * *p* < 0.05, Wilcoxon signed-rank test). The black circle symbols represent the data for individual neurons. The effect of intracellular γ-cyclodextrin on the mIPSC decay time of individual paired recordings is shown by the line connecting the black closed symbols of the two columns. γ-CD (γ-cyclodextrin).

There is now convincing evidence that the endogenous levels of the GABA_A_R-active neurosteroids, such as allopregnanolone, are sufficient to influence neural inhibition [43,44]. The cyclic sugar structure of γ-cyclodextrin sequesters lipophilic molecules, including neurosteroids [45,46]. By including γ-cyclodextrin in the recording pipette, we demonstrated that for neonatal thalamic and cortical neurons, phasic inhibition is enhanced by an endogenous neurosteroid tone [47,48]. Here, for adult MSNs, the inclusion of γ-cyclodextrin in the recording pipette produced a significant reduction in the mIPSC decay time during the course of the recording, with the mIPSCs recorded > 180 s after achieving the whole-cell configuration (i.e., dialysing the cell content with the intracellular solution including γ-cyclodextrin) being significantly faster (6.4 ± 0.5 ms, n = 6 neurons) in comparison to those events recorded during the first 120 s (7.9 ± 0.9 ms, n = 6 neurons, *p* < 0.05 Wilcoxon signed-rank test)—(Figure 5). This observation suggests the presence of a neurosteroid tone for adult MSNs, sufficient to influence GABA_A_R signalling, but does not identify whether the steroid is produced in the accumbens to then act in a local paracrine or autocrine manner.

**Figure 6:**
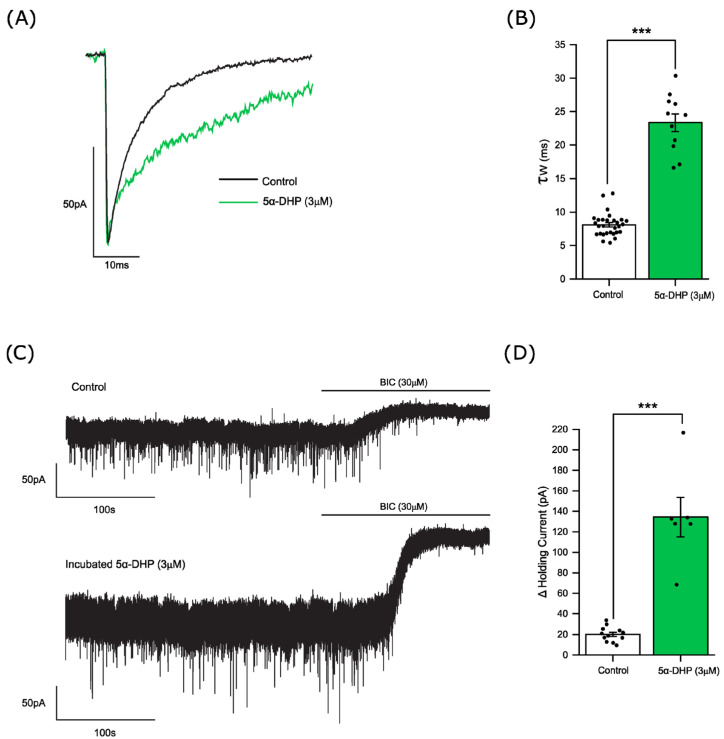
Putative synthesis of a GABA_A_R-active steroid by adult mouse nucleus accumbens. The effect of 5α-pregnanedione (3 µM preincubation > 3 h followed by bath perfusion) upon phasic and tonic inhibition of accumbal MSNs. (**A**) Superimposed averaged mIPSCs recorded under control (black trace) and following 5α-pregnanedione treatment (green trace). (**B**) A histogram illustrating that 5α-pregnanedione (3 µM) significantly prolonged the mIPSC decay time constant (control τ_w_ = 8.1 ± 0.3 ms, n = 29 neurons; 5α-pregnanedione τ_w_ = 23 ± 1.3 ms; n = 11 neurons *** *p* < 0.001 *c.f.* control; independent samples *t*-test). Each column represents the mean ± SEM of the mIPSC decay time with the value for individual neurons denoted by the closed black circle (**C**) Exemplar recordings illustrating the effect of bicuculline (30 µM) on the holding current of a control MSN (top trace) and an MSN treated with 5α-pregnanedione. (**D**) A histogram illustrating the mean change ± SEM of the holding current for control and 5α-pregnanedione treated neurons (control = 20 ± 2.1 pA, n = 12 neurons; 5α-pregnanedione = 134 ± 19.3 pA, n = 6 neurons; *** *p* < 0.001 *c.f.* control; Mann- Whitney U test). The closed black circle represents the result for individual neurons. BIC = bicuculline, 5α-DHP = 5α-pregnanedione.

To investigate a putative steroidogenic capacity of the accumbens, we determined the influence of 5α-pregnanedione, the immediate precursor of allopregnanolone, on accumbal GABA-ergic transmission. In contrast to allopregnanolone, 5α-pregnanedione does not act as a direct PAM of the GABA_A_R [47,48]. However, we reported that preincubation of mouse thalamic and cortical slices with 5α-pregnanedione resulted in a prolongation of the mIPSCs, an effect prevented by co-incubation with the 3α-hydroxy steroid dehydrogenase inhibitor indomethacin [47,48]. Here, incubation (~3 h) of accumbal slices with 5α-pregnanedione (3 µM) greatly prolonged the mIPSC decay time (control τ_w_ = 8.1 ± 0.3 ms, n = 29 neurons, pregnanedione τ_w_ = 23 ± 1.3 ms; n = 11 neurons, *p* < 0.001 independent samples *t*-test) and produced a large increase in the tonic current (control = 20 ± 2.1 pA, n = 12 neurons; 5α-pregnenedione = 134 ± 19 pA; n = 6 neurons *p* < 0.001 Mann–Whitney U test)—(Figure 6A,B). Collectively, these findings suggest that the accumbal slice can metabolise 5α-pregnanedione to the GABA_A_R active PAM allopregnanolone.

The profile of neurosteroids and synthetic neuroactive steroids is of potential therapeutic interest.

### 3.6. Sage-217 (Zuranolone)

**Figure 7:**
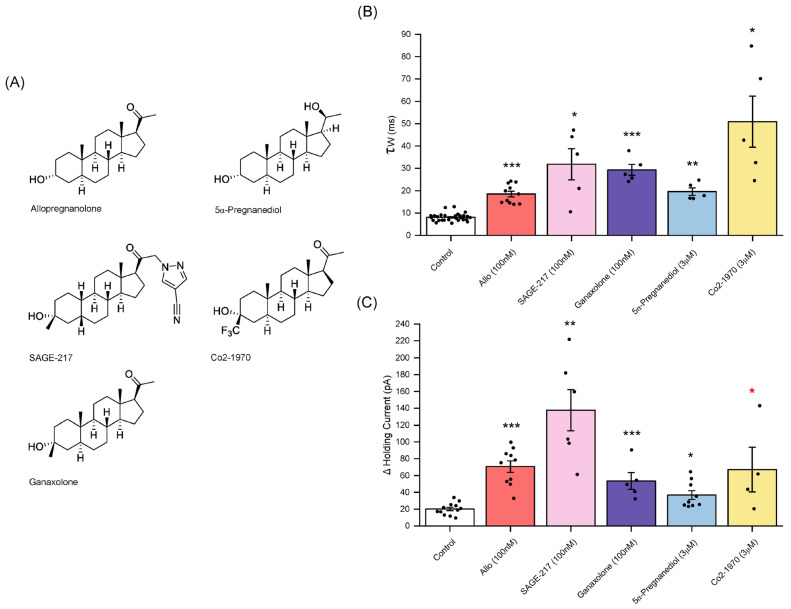
The effect of neurosteroids and synthetic neuroactive steroids on phasic and tonic inhibition of mouse accumbal medium spiny neurons. (**A**) The structures of the steroids investigated. (**B**,**C**) Histograms illustrating the effect of allopregnanolone (100 nM), SAGE-217 (100 nM), ganaxolone (100 nM), 5α-pregnane-3α,20α-diol (3 µM) [5α-pregnanediol] and Co2-1970 (3 uM) on (**B**) the mIPSC decay (τ_w_ ms) and (**C**) the bicuculline (30 µM)-induced change in the holding current (pA). All steroids tested produced (**B**) a significant prolongation of the mIPSC decay time and (**C**) an increased holding current. (**B**,**C**) Each column represents the mean ± SEM with the effect for individual neurons denoted by the black circle. *** *p* < 0.001 ** *p* < 0.01 * *p* < 0.05 *c.f.* control; independent samples *t*-test; * *p* < 0.05 compared to control, Mann–Whitney U test).

Zuranolone has recently been approved by the Food and Drug Administration (FDA) to treat PPD [2,4]. Using an equivalent concentration to allopregnanolone, an >2 h incubation of SAGE-217 (100 nM) produced a significant prolongation of the mIPSCs (control τ_w_ = 8.1 ± 0.1 ms; n = 29 neurons, Sage-217 τ_w_ = 32 ± 7.0 ms n = 5 neurons, *p* < 0.05 independent samples *t*-test) and a significant large increase in the tonic current, revealed by 30 µM bicuculline (control = 20 ± 2.1 pA, n = 12 neurons; Sage-217 = 138 ± 25 pA, n = 6 neurons, *p* < 0.01, independent samples *t*-test)—Figure 7.

### 3.7. Ganaxolone

Here, in common with allopregnanolone, preincubated (>2 h) ganaxolone (100 nM) significantly prolonged the mIPSC decay time (control τ_w_ = 8.1 ± 0.1 ms, n = 29 neurons, ganaxolone τ_w_ = 29 ± 2.5 ms, n = 5 neurons, *p* < 0.001, independent samples *t*-test) and increased the tonic current, as revealed by bicuculline (control = 20 ± 2.1 pA; n = 12 neurons; ganaxolone = 53 ± 10 pA; n = 5 neurons; *p* < 0.001, independent samples *t*-test)—Figure 7.

### 3.8. 5α-pregnan-3α,20α-diol

5α-*pregnan-3α,20α-diol* (5α-pregnanediol) is a major metabolite of progesterone, with a behavioural profile distinct from allopregnanolone (see Discussion). Although it acts as a PAM of GABA_A_Rs, both radioligand binding and electrophysiological studies reveal 5α-pregnanediol to exhibit reduced efficacy in comparison to allopregnanolone, a property that may underpin the distinctive behavioural profile of this steroid [49,50,51,52,53]—see Section 4. Although electrophysiological studies with recombinant GABA_A_Rs demonstrated efficacy at nM concentrations, here for neuronal GABA_A_Rs preliminary experiments revealed low µM concentrations were required to produce a clear effect on tonic inhibition. Specifically, preincubated (>2 h) 5α-pregnanediol (3 µM) significantly, albeit modestly, increased the tonic current vs. control as revealed by bicuculline (37 ± 5 pA; n = 9 neurons *p* < 0.05, independent samples *t*-test) but produced a large prolongation of the mIPSCs (control τ_w_ = 8.1 ± 0.3 ms; n = 29 neurons; 5α-pregnanediol τ_w_ = 19.6 ± 1.7 ms; n = 5 neurons; *p* < 0.01, independent samples *t*-test) similar to that produced by allopregnanolone 100 nM—Figure 7.

### 3.9. Co2-1970 (3α-Hydroxy-3β-Trifluromethyl-5α-Pregnan-20-One)

Previous studies reported the synthetic steroid Co2-1970 (0.1–1µM) to enhance the GABA-evoked current mediated by recombinant GABA_A_Rs (α1β1γ2) expressed in *Xenopus* oocytes, although with reduced efficacy when compared to allopregnanolone [54]. For comparison with 5α-pregnanediol we investigated the effect of Co2-1970 (3 µM) on phasic and tonic inhibition. Preincubated (>2 h) Co2-1970 produced a large prolongation of the mIPSC decay (control τ_w_ = 8.1 ± 0.3 ms; n = 29 neurons; Co2-1970 τ_w_ = 51 ± 12 ms; n = 5 neurons; *p* < 0.05, independent samples *t*-test) and a significant increase in the tonic current (control = 20 ± 2.1 pA; n = 12 neurons; Co2-1970 = 67 ± 27 pA; n = 4 neurons, *p* < 0.05, Mann–Whitney U test)—Figure 7.

## 4. Discussion

The FDA approval of allopregnanolone (brexanolone) and the synthetic derivative SAGE-217 (zuranolone) to treat PPD has further encouraged targeting GABA_A_R isoforms to develop novel therapeutics for major depressive disorders [2,3,4,5,6,7,8,9,11]. In this regard, extrasynaptic GABA_A_Rs incorporating the δ-subunit have received particular attention, with much of the focus centred on their role in hippocampus and amygdala. By contrast, the VTA–nucleus accumbens circuit of the mesolimbic system has received limited consideration. This pathway comprises an important component of the reward system and is implicated in disrupted mood and anhedonia, both of which are integral components of depressive disturbances [12,13,14]. Importantly, changes in the activity of this pathway are implicated in PPD [15,16].

### 4.1. The Tonic Current Is Mediated by Spontaneously Open GABA_A_Rs

Mouse accumbal core MSNs exhibit fast phasic inhibition mediated by synaptic GABA_A_Rs composed of α1βγ2- and α2βγ2 subunits and tonic inhibition mediated by extrasynaptic GABA_A_Rs composed of α4, β, and δ subunits, respectively [25,26,27]. We investigated the effects of GABA-ergic steroids on phasic and tonic inhibition of accumbal MSNs, with a particular focus on the latter. The GABA_A_R antagonist bicuculline abolished the mIPSCs mediated by synaptic GABA_A_Rs and inhibited the tonic current. In common, gabazine also inhibited the mIPSCs but in contrast had no effect on the holding current. Bicuculline binds to the orthosteric site and acts as a negative allosteric modulator (NAM) of the GABA_A_R, causing a conformational change to promote channel closure, whereas although gabazine also binds to the orthosteric site, it acts as a competitive antagonist of the transmitter GABA [33,34,35,36,37]. The differential effect of the antagonists suggests that the resident tonic current is mediated by spontaneously open GABA_A_Rs and furthermore implies that the ambient levels of GABA are insufficient to gate these extrasynaptic GABA_A_Rs. In support, following gabazine, the application of picrotoxin, a non-competitive GABA_A_R channel blocker, revealed an outward current. An alternative explanation posits that the extrasynaptic receptors (α4βδ) are gabazine-insensitive, although that is unlikely as recombinant α4βδ GABA_A_Rs expressed in cell lines are inhibited by gabazine [55].

The resident tonic current of hippocampal dentate gyrus granule neurons is also gabazine-insensitive, but this antagonist becomes effective in the presence of added GABA [37]. Furthermore, single channel recordings from nucleated patches of these granule cells revealed gabazine-insensitive spontaneous GABA_A_R channel openings [37].

Studies of recombinant δ-GABA_A_Rs incorporating the α4 or the α6 but not α1 subunit isoform exhibit GABA-independent gating [35]. Additionally, the β-subunit isoform is crucial in governing spontaneous activity. When expressed in cell lines, GABA_A_Rs composed of α4, β3, and δ subunits exhibited spontaneous activity, whereas equivalent receptors incorporating the β1 or β2 subunit did not [35]. This selectivity is influenced in part by a four amino acid motif in the extracellular domain of the β3 subunit and the phosphorylation status of β3 serine residues located within the large intracellular loop linking the TM3 and TM4 transmembrane regions. These serine residues are substrates for certain protein kinases, including PKA and PKC [35]. The spontaneous gating of extrasynaptic GABA_A_Rs of dentate gyrus neurons is reduced by PKC inhibition and by conditions known to influence kinase activity including temperature (34 °C vs. 24 °C), and levels of intracellular calcium chelators (favoured by low 50 µM vs. high mM intracellular EGTA) [36]. Here, our studies on accumbal MSNs were performed at 34 °C, but with relatively high (10 mM) intracellular EGTA. In common with dentate, in preliminary experiments the resident tonic current of MSNs as revealed by bicuculline was reduced by the PKC inhibitor (20 µM) GF109203X (GFX). Our immunohistochemistry reveals the expression of β1 and β3 subunits in the accumbal MSNs. The demonstration of GABA-independent gating of the resident tonic current suggests a population of extrasynaptic receptors expressed in these neurons that incorporate the β3 subunit. Clearly, the recording conditions can influence the degree of spontaneous gating.

### 4.2. The Influence of Allopregnanolone on Phasic and Tonic Inhibition

Incubation of allopregnanolone (100 nM) greatly prolonged the duration of mIPSCs mediated by synaptic GABA_A_Rs and produced a large increase in the bicuculline-sensitive tonic current. However, in the presence of the neurosteroid, a substantial element of the tonic current was now gabazine-sensitive, although a component remained insensitive as revealed by picrotoxin. The binding of allopregnanolone is proposed to enhance the interaction of GABA with GABA_A_Rs by inducing an allosteric rearrangement of the GABA-binding site located in the extracellular domains [43]. This change in the antagonist pharmacology suggests that the neurosteroid enhanced the action of ambient GABA sufficiently to now gate the extrasynaptic receptors. In support, whereas gabazine has no effect on the tonic current of dentate gyrus granule cells, it does so in the presence of a low concentration of GABA [37]. We reported that the tonic current of accumbal MSNs was greatly reduced by deletion of either the δ or the α4 subunit, although a small tonic current remained, mediated by an unidentified GABA_A_R subtype [25]. Given that allopregnanolone is not selective for δ-GABA_A_Rs [42], the introduction by the steroid of a gabazine-sensitive component to the tonic current cannot be assumed to be mediated solely or in part by δ-GABA_A_Rs. However, in common with allopregnanolone, a fraction of the increased tonic current produced by DS2, a δ-GABA_A_R-selective PAM [41], was also gabazine-sensitive. Collectively, these observations suggest that allopregnanolone increases the tonic current by enhancing the effect of ambient GABA, such that it now gates the α4βδ GABA_A_R receptor.

### 4.3. Evidence for an Endogenous Neurosteroid Tone and Local Neurosteroid Synthesis in the Nucleus Accumbens

There is accumulating evidence that the endogenous level of neurosteroids, such as allopregnanolone, are sufficient to influence GABA-ergic transmission. A recent cryo-EM coupled with mass spectrometry study of mouse brain GABA_A_Rs reported isolated native receptors to be occupied by endogenous allopregnanolone [43]. Studies of a mouse engineered to carry a point mutation on the α2 subunit (α2Q241M) that imparts neurosteroid-insensitivity have been informative [44]. Behaviourally, such mice exhibited an anxious phenotype and the anxiolytic effect of systemically injected neurosteroid was impaired. Voltage-clamp recordings of dentate gyrus granule cells of the α2Q241M mouse exhibit mIPSCs of reduced duration and the effect of exogenous neurosteroid to prolong phasic inhibition is reduced [44]. Collectively, these findings imply the levels of endogenous neurosteroids are sufficient to influence GABAergic transmission and behaviour. Employing the steroid scavenger γ-cyclodextrin we demonstrated GABA-ergic neurotransmission of neonatal mouse thalamic and cortical neurons to be influenced by a neurosteroid tone [47,48]. In these studies, the effect of γ-cyclodextrin was negated by prior treatment with indomethacin, an inhibitor of the 3α-hydroxysteroid dehydrogenase enzyme required to convert 5α-pregnanedione to the GABA_A_R-active steroid allopregnanolone [47,48]. Here, similarly, the inclusion of γ-cyclodextrin in the recording pipette produced a reduction in the mIPSC decay time of adult mouse MSNs during the first few minutes of recording, reflecting the dynamic incorporation of the endogenous neurosteroid by γ-cyclodextrin.

The levels of allopregnanolone in rat nucleus accumbens increase following novelty-induced stress (exploration in the open field test) [56]. Furthermore in situ hybridisation reveals the mouse striatum to express the mRNAs encoding for the 5α-reductase and 3α-HSD enzymes required to synthesise allopregnanolone [57]. However, the synthesis of the steroid, suggested by the γ-cyclodextrin experiment, cannot be assumed to be local to the nucleus accumbens. We reported that preincubation of neonatal thalamic slices with the allopregnanolone precursor 5α-pregnanedione produced a prolongation of the mIPSC decay time mediated by α1βγ2 synaptic GABA_A_Rs, and an increased tonic current mediated by α4β2δ extrasynaptic GABA_A_Rs, effects reduced by co-incubation with the 3α-HSD inhibitor indomethacin [47]. Here, prolonged incubation of the adult accumbal slice with 5α-pregnanedione produced a substantial prolongation of phasic inhibition and a large increase in tonic inhibition. Collectively, these observations suggest the adult accumbens has the steroidogenic capacity to synthesise GABA_A_R-active neurosteroids.

### 4.4. The Profile of Neurosteroids and Synthetic Neuroactive Steroids of Potential Therapeutic Interest

Following the FDA endorsement of allopregnanolone (brexanolone) to treat PPD, the synthetic neuroactive steroid Sage-217 (zuranolone) has now also been approved for use in this depressive disorder [2]. Brexanolone requires i.v. infusion over 60 h in a hospital setting. Importantly, in contrast to brexanolone, zuranolone is active when given orally, thereby overcoming some of the complications associated with *i.v.* administration of brexanolone.

The impact of acute allopregnanolone (100 nM) on both phasic and tonic inhibition was greatly increased by a preincubation protocol. The effect to prolong the mIPSCs suggests preincubation improved access of the steroid to the neuron within the slice, thereby enhancing the affinity of GABA for synaptic GABA_A_Rs and extrasynaptic δ-GABA_A_Rs [39,40]. Potentially complementing this effect, the steroid may increase the cell surface expression and/or enhance the spontaneous gating of the extrasynaptic α4β3δ GABA_A_Rs. Phosphorylation of the β3 subunit S408/409 residues plays a key role in both the facilitation of spontaneous gating and the increased cell surface receptor expression [35,58,59,60,61]. Indeed, allopregnanolone had no effect on the tonic current of dentate granule cells obtained from a phosphorylation-resistant “knock-in” mouse expressing a β3 subunit 408/9 serine to alanine mutation [59]. Furthermore, in behavioural studies, the anxiolytic and anticonvulsant effects of neuroactive steroids were impaired in these mice [62]. In the accumbens, our observation that following steroid incubation the effect of gabazine to now inhibit a component of the allopregnanolone-enhanced tonic current appears incompatible with the steroid acting primarily to increase spontaneous gating of the receptor in these accumbal neurons. However, increased extrasynaptic receptor expression by incubated allopregnanolone may contribute to the augmented tonic current. This effect of allopregnanolone on trafficking of α4β3δ GABA_A_Rs to the cell surface is reported to result from the steroid activating a G-protein coupled progesterone receptor (mPR) [58,59]. To support this finding, members of the mPR progesterone receptor family are expressed in the human nucleus accumbens [63].

*Ganaxolone:* Ganaxolone is a 3β-methyl analogue of allopregnanolone. This modification impairs the enzymic metabolism of the 3α-hydroxyl group, critical for GABA_A_R activity [10]. Consequently, ganaxolone has a longer half-life than allopregnanolone, permitting oral administration. Additionally, allopregnanolone but not ganaxolone can be converted back to 5α-pregnanedione. This latter steroid is genomically active, including binding to progesterone receptors, whereas ganaxolone is inert in this respect [10]. Ganaxolone is currently undergoing clinical trials as a putative treatment for PPD [4]. Additionally, ganaxolone (ZTALMY) was recently approved as an anticonvulsant to treat seizures associated with cyclin-dependent kinase-like 5 deficiency disorder (CDKL5), is in trials to treat tuberous sclerosis complex-related epilepsy, and is being assessed in refractory status epilepsy [64]. Here, in common with allopregnanolone (100 nM), ganaxolone (100 nM) produced a substantial prolongation of phasic inhibition mediated by synaptic GABA_A_Rs and a large increase in the tonic current mediated by extrasynaptic GABA_A_Rs. In the VTA, acute ganaxolone (500 nM) had little effect on spontaneously occurring IPSCs (sIPSCs) of either the GABA or dopamine neurons, or the tonic current of the dopamine neurons, but preferentially enhanced the tonic current of the GABA-ergic interneurons [20]. For mouse dentate gyrus granule neurons acute ganaxolone (100 nM) increased the tonic current and prolonged the phasic current [58].

*5α-pregnan-3α,20α-diol (5α-pregnanediol)* is a major metabolite of progesterone, with raised levels evident during pregnancy [49,50,51]. Sedation and indeed in some cases unconsciousness are reported side effects associated with allopregnanolone (brexanolone) treatment of PPD [8]. In this context, the endogenous neurosteroid 5α-pregnanediol is of interest. In rats this steroid exhibits anxiolytic effects, at lower doses than those required to produce motor deficits [49,50,51,52,53]. This distinctive profile may relate to the limited GABA_A_R efficacy reported for this steroid in radioligand binding assays and electrophysiological functional studies [49,50,51]. In comparison to allopregnanolone (100 nM), a greater concentration (3 µM) of 5α-pregnanediol, was required to enhance the tonic current of MSNs, but surprisingly this concentration, albeit 30-fold greater, appeared as effective as allopregnanolone (100 nM) in prolonging the mIPSC decay, suggesting a selective interaction of 5α-pregnanediol with synaptic vs. extrasynaptic GABA_A_Rs of accumbal MSNs. In this respect, it is intriguing that recent studies employing a steroid photoaffinity radiolabel and human recombinant GABA_A_Rs suggest a pregnanediol binding site distinct from that of allopregnanolone [65]. Thus, both the limited efficacy profile and a different site of action on the receptor may contribute to the distinct behavioural profile compared to allopregnanolone. Such a profile may also be physiologically relevant. Thus, for example, 5α-pregnanediol levels are raised in pregnancy [66] and in catamenial epilepsy; levels of this steroid are inversely correlated with the incidence of seizure [49,67]. Collectively, these observations suggest that neuroactive steroids with a profile akin to that of 5α-pregnanediol may offer a route to develop new therapeutics with a more limited propensity for sedation.

*Co2-1970 (3α-Hydroxy-3β-Trifluromethyl-5α-Pregnan-20-One):* As described above, the structure of ganaxolone is a modified allopregnanolone with the addition of a 3β-methyl group to protect from metabolism the 3α-hydroxyl group, crucial for GABA_A_R activity, thereby improving the pharmacokinetic profile of the steroid. Adopting this strategy, Co2-1970 is allopregnanolone with the addition of a trifluoromethyl group in the 3β position. The addition of the 3β-methyl group had little effect on the GABA_A_R potency and efficacy of ganaxolone in comparison to allopregnanolone, Figure 7. By contrast, for Co2-1970 the 3β-trifluoromethyl substitution reduced both the potency and efficacy of the steroid [54]. Additionally, Co2-1970 reduced the GABA_A_R-enhancing effect of allopregnanolone, implying that this steroid behaves as a partial GABA_A_R PAM [54]. For accumbal MSNs, Co2-1970 (3 µM) produced an enhancement of the tonic current similar to that produced by allopregnanolone (100 nM), but greatly prolonged the mIPSCs, suggesting as for 5α-pregnanediol, a preferential interaction with synaptic GABA_A_Rs. The latter is of interest given the evidence that these synaptic GABA_A_Rs are influenced by a resident neurosteroid tone (Figure 5) and the effect of Co2-1970 to reduce the GABA-modulatory effects of allopregnanolone [54]. It would be of interest to explore the behavioural effects of Co2-1970 given the separation of the anxiolytic and sedative effects of 5α-pregnanediol [50,52,53].

In conclusion, we are entering an exciting era of identifying new molecular targets to develop novel treatments for depressive disorders that have a more rapid onset of action and are effective in a greater proportion of patients [68]. For the neuroactive steroids and PPD, further studies are required to improve understanding of the plasticity and target(s) underpinning the reported prolonged clinical benefit that outlasts steroid treatment [4,7]. Studies with mice genetically engineered to lack or limit expression of the δ-subunit protein suggest that δ-GABA_A_Rs make an important contribution to antidepressant efficacy of the steroids [69]. The development of selective δ-GABA_A_R PAMs that readily cross the blood–brain barrier would be instructive in assessing the role of these receptors. In this regard, the recent report of a brain-penetrant, potent, selective δ-GABA_A_R inhibitor is of interest in permitting a better understanding of the role of these extrasynaptic receptors [70].

## Data Availability

The data presented in this study are available in the main text, figures, tables.
